# Association between Alcohol Consumption and Body Composition in Russian Adults and Patients Treated for Alcohol-Related Disorders: The Know Your Heart Cross-Sectional Study

**DOI:** 10.3390/ijerph20042905

**Published:** 2023-02-07

**Authors:** Nikita A. Mitkin, Tatiana N. Unguryanu, Sofia Malyutina, Alexander V. Kudryavtsev

**Affiliations:** 1 Department of Community Medicine, UiT The Arctic University of Norway, N-9037 Tromsø, Norway; 2 International Research Competence Centre, Northern State Medical University, Troitsky Ave., 51, 163069 Arkhangelsk, Russia; 3 Department of Hygiene and Medical Ecology, Northern State Medical University, Troitsky Ave., 51, 163069 Arkhangelsk, Russia; 4 Research Institute of Internal and Preventive Medicine, Branch of Institute of Cytology and Genetics, Siberian Branch of the Russian Academy of Sciences, Bogatkova st., 175/1, 630008 Novosibirsk, Russia; 5 Department of Therapy, Hematology and Transfusiology, Novosibirsk State Medical University, Krasny Ave., 52, 630091 Novosibirsk, Russia

**Keywords:** alcohol, body composition, body fat percentage, body mass index, waist circumference, hip circumference, waist-to-hip ratio

## Abstract

There is conflicting evidence about the association between alcohol consumption and body composition (BC). We aimed to investigate this association in Russian adults. The study population included 2357 residents of Arkhangelsk aged 35–69 years, and 272 in-patients treated for alcohol problems (narcological patients) who participated in the Know Your Heart (KYH) cross-sectional study in 2015–2017. The participants were divided into five subgroups based on their alcohol use characteristics: non-drinkers, non-problem drinkers, hazardous drinkers, harmful drinkers, and narcological patients. Considering men, hazardous drinkers had a larger waist circumference (WC), waist-to-hip ratio (WHR), and percentage of body fat mass (%FM) compared to non-problem drinkers. In harmful drinking men, these differences were the opposite: a lower body mass index (BMI), hip circumference (HC), and %FM. Men among narcological patients had the lowest mean BMI, WC, HC, WHR, and %FM compared to other subgroups of men. As for women, non-drinkers had a lower BMI, WC, HC, and %FM compared to non-problem drinkers. Women among narcological patients had the lowest mean BMI and HC but an increased WHR compared to other subgroups of women. In conclusion, alcohol consumption levels had an inverted J-shaped association with adiposity-related BC parameters: they were elevated in hazardous drinkers but were reduced in harmful drinkers, and were even lower in patients with alcohol-related diagnoses.

## 1. Introduction

Alcohol, or ethanol, has been a part of almost every human culture for thousands of years [1,2]. Today, nearly one-third of the world’s population consumes alcoholic beverages regularly. However, excessive alcohol use has significant negative impacts on overall health, leading to over 200 different diseases, and is one of the leading causes of death globally, resulting in 3 million deaths annually [3].

In recent years, global per capita alcohol consumption has increased and is projected to reach 7.6 L by 2030 [4]. Europe remains the region with the highest per capita alcohol intake, with an average level of 9.8 L in 2016 [3]. Excess ethanol consumption in Europe has the highest attributable health burden, causing every tenth year of life lost prematurely [5]. According to an Eastern European population study, drinking above 60 g/day among men and drinking above 20 g/day among women increased the risk of all-cause mortality by 23% and 92%, respectively [6].

Historically, Russia had a higher than global per capita alcohol consumption level, reaching 18.7 L in 2005 [3]. The implementation of national alcohol control policies in the mid-2000s gradually reduced the annual consumption of pure ethanol to 11.7 L per capita in 2016 [3,7]. This contributed to a reduction in all-cause mortality and an increase in life expectancy [7,8]. Despite these improvements, the proportion of alcohol-attributable deaths in Russia remained higher than the global average by 13.8% for men and 4.5% for women [9].

Excess alcohol drinking continues to be a major public health concern in Russia [10]. In 2016, the annual alcohol consumption was 20% higher compared to the European region. In the same year, 80% of men and 64% of women in Russia were current drinkers, with an average daily level of pure alcohol consumption of 50 mL for men and 20 mL for women [3]. In the most recent two decades, drinking vodka and wine have been partly replaced by beer with a parallel reduction in heavy binge drinking [11]. Nevertheless, the Russian drinking pattern is still characterized by the widespread use of spirits, which are consumed in large amounts per occasion [3].

The effects of alcohol on health are influenced by various factors, including the amount, frequency, and type of alcoholic beverage consumed [3,12]. While the harmful health effects are broadly described [3,9,13,14], the evidence of alcohol associations with body composition (BC) parameters remains lacking [15,16,17]. Ethanol is a known energy-dense macronutrient and produces 7.1 kilocalories per gram (or ~29 kJ/g), which is second after fats. It cannot be stored within the body, suppresses fat oxidation, and increases fat storage [18]. In addition, alcohol drinking is associated with enhanced appetite [19] and an increase in daily caloric intake of about 10% [20]. In line with this, several studies have demonstrated a positive association between alcohol consumption and body mass index (BMI) [15,16,17,21]. Other studies have shown a null or negative association [18,22,23,24]. These conflicting findings reflect the need for further research of the effects of alcohol intake on BC, particularly in the light of the growing pandemic of obesity [25,26,27].

In this study, we investigated the associations between alcohol consumption and BC parameters in a Russian adult population using a general population sample and a group of in-patients treated for alcohol-related disorders.

## 2. Materials and Methods

### 2.1. Study Populations

The study population comprised men and women aged 35–69 years who participated in the Know Your Heart (KYH) cross-sectional study, conducted in 2015–2017 as part of the International Project on Cardiovascular Disease in Russia (https://knowyourheart.science/). The KYH study included a random sample from the general population of Arkhangelsk (N = 2381) (main study). In addition, 275 in-patients treated for alcohol-related diagnoses were recruited in the same city (narcological sub-study). A detailed description of the KYH study rationale, design, and methodology was published previously [28].

### 2.2. Recruitment of Participants

Participants of the main study were recruited using an anonymized address database of the Arkhangelsk regional health insurance fund, supplemented by age and sex information of the insured living at an address. The sampling was stratified by age, sex, and city district. Trained interviewers visited randomly selected addresses up to five times to make contact. When contact was established, the targeted household members were identified by age group and sex and invited to participate in the study. Those who agreed had a baseline interview at home and were offered a health check at the polyclinic of the Northern State Medical University (Arkhangelsk). Among all the invitees, the response rate was 68%. Of the total interviewed, 96% took part in the health check.

Participants of the narcological sub-study were recruited from in-patients with primary diagnoses of alcohol-related disorders at the narcology departments of the Arkhangelsk Regional Psychiatric Hospital. Physicians invited patients who had a documented history of alcohol abuse (at least five years of problem drinking) and were in current in-patient treatment for at least a week after admission. Patients were excluded if they had behavioral problems that could pose a threat to the safety of study staff and other participants, if they used illicit drugs, or if they were unable to provide informed consent (e.g., because of severe cognitive deficits or acute mental illness). Patients who met the criteria and agreed to participate had the baseline interview at the hospital and were transported to the polyclinic for the health check. The response rate in this group was 85%.

In this study, we used data on 2629 KYH study participants: 2357 participants from the main study in Arkhangelsk and 272 participants from the narcology subsample who had completed the baseline interview and the health check, answered questions on alcohol consumption, and undergone BC measurements.

### 2.3. Data Collection

Baseline interviews were conducted by trained interviewers using Computer-Assisted Personal Interview (CAPI) tablets. They collected data on participants’ demographic and socio-economic characteristics, lifestyle, and medical history. The interview included questions on alcohol consumption in the last 12 months: (1) frequency and usual quantity of drinking alcoholic beverages (for assessments of annual ethanol consumption using the quantity–frequency approach [29]), (2) the Cutting down, Annoyance by criticism, Guilty feeling, and Eye-openers (CAGE) questionnaire for screening alcohol abuse [30,31], (3) seeking medical care because of alcohol-related problems, and (4) attributes of alcohol abuse: frequency of “zapoi” episodes (an alcohol drinking period of two or more days, during which a person falls out of a routine social life), hangover, excessive drunkenness, sleeping in clothes at night, and failing to fulfill family or other obligations due to drunkenness. For the main study participants, but not for the narcological patients, the interview also collected data on physical activity using the EPIC questionnaire [32,33,34] and on dietary intake using the Dietary Quality Score questionnaire [35].

Health check included physical examination, collection of blood samples, and a medical interview, conducted by trained physicians and nurses. The interview comprised questions on history and symptoms of diseases, medication use, and health behaviors, and included Alcohol Use Disorders Identification Test (AUDIT) [36,37,38,39]. Physical examination comprised instrumental and functional measurements of cardiovascular health, including anthropometric and BC parameters.

Blood samples were taken at least four hours after the last meal, centrifuged with serum aliquoted into cryovials, frozen and stored at −80 °C. At the end of the fieldwork, the samples were shipped on dry ice to a laboratory in Moscow, where they were analyzed. Serum level of carbohydrate-deficient transferrin (CDT, %), a marker of ethanol consumption, was measured for a subsample of 1242 KYH participants (976 from the main study sample and 266 narcological patients) by capillary electrophoresis (CAPILLARYS-2 automatic capillary electrophoresis system, Sebia S.A., France).

### 2.4. Outcome Variables

Body composition was assessed using anthropometric and bioelectrical impedance measurements [40]. Height (cm), hip circumference (HC, cm), and waist circumference (WC, cm) were measured using Seca^®^ 217 portable stadiometer (Seca Limited) and Seca measuring tapes (Seca^®^ 201) (Seca Limited), respectively. Weight (kg) and the percentage of body fat mass (%FM) were measured using TANITA BC-418 Body Composition Analyzer (TANITA, Europe GmbH) [41]. At the measurement, the participants were barefoot, were wearing light clothing, and had all metal objects, such as jewelry and watches, removed. Participants of the KYH study with cardio stimulators were not examined using TANITA and were not included in this study. Body mass index (BMI) was calculated as weight (kg) divided by height squared (m^2^). Waist-to-hip ratio (WHR) was calculated as the ratio of waist circumference to hip circumference.

### 2.5. Exposure Variable

The alcohol exposure variable was derived from several alcohol-related characteristics assessed in the KYH study. First, the study participants were by default split into the general population KYH sample (the main study) and the additional sample of narcological patients. Second, we categorized the main study participants into four groups: non-drinkers, non-problem drinkers, hazardous drinkers, and harmful drinkers.

Non-drinkers were defined as those who reported no alcohol intake in the past 12 months in both baseline and medical interviews. Those who reported alcohol use in the past 12 months and had an AUDIT score < 8 and a CAGE score < 2 were classified as non-problem drinkers. The category of hazardous drinkers included those who reported alcohol use in the past 12 months and had an AUDIT score of 8–15 and/or a CAGE score of 2–3. Participants were defined as harmful drinkers if they reported alcohol use in the past 12 months and had at least one of the following characteristics: AUDIT score of 16 or higher [36,37,38,39]; CAGE score of 4 [30,31]; self-reporting one or more episodes of “zapoi” in the past 12 months; self-reporting hangover and/or excessive drunkenness and/or sleeping in clothes at night and/or failing to fulfill family or other obligations because of drunkenness two or more times per week. The derived categorical variable was used as a measure of alcohol exposure in all analyses.

Previously, Iakunchykova et al. applied a similar categorization of the KYH study population according to the level of alcohol exposure [42]. They demonstrated that the identified groups differed in the total volume of ethanol consumed during the last year, in self-reported medical treatment for alcohol problems during the last year, and in blood levels of biomarkers of excessive alcohol consumption (γ-glutamyltransferase (GGT) and CDT) [43]. This categorization approach was described as valid because it was based on several alcohol-related characteristics, thus minimizing recall bias compared with one-measurement-based indicators, such as total ethanol intake per year estimated using the frequency–quantity method.

In this study, to substantiate the division of participants into groups by alcohol consumption levels, we also compared the studied groups on several alcohol-related variables: AUDIT score categories (<7; 8–15; 16–19; >20) [36], frequency of drinking alcohol (nearly every day, 1–5 times a month, a few times a year, or never); frequency of drinking alcohol in the past 12 months by three types of beverages (beer, wine, and spirits); amount of alcohol consumed per occasion by the three types of beverages; estimated total annual amount of alcohol consumed by the three types of beverages [29]; and the CDT level in blood serum [43].

### 2.6. Covariates

Other KYH study variables used in this study as potential confounders were age (years), education level (primary or incomplete secondary, secondary, higher), smoking status (never smoker, ex-smoker, current smoker), and presence (yes/no) of chronic non-communicable diseases (NCD) that could be associated with alcohol consumption and BC parameters. We considered NCDs present if the participant self-reported any of the listed: cardiovascular disease (hypertension, angina, myocardial infarction, stroke, heart failure, or atrial fibrillation), cancer, chronic liver disease, or chronic kidney disease.

For the main study participants, but not for narcological patients, we had additional data on physical activity and dietary quality. For analyses, physical activity (assessed using the EPIC questionnaire [32,33,34,44]) was categorized into four levels (inactive, moderately inactive, moderately active, and active). Diet (assessed with the Dietary Quality Score [35]) was analyzed as a variable with three categories (unhealthy, average, healthy).

### 2.7. Statistical Analysis

All analyses were stratified by gender. We used absolute numbers and relative frequencies to present categorical variables. Means (M) with standard deviations (SD) or medians (Me) with 25th and 75th percentiles (P25; P75) were used to present continuous variables, depending on their distributions. The studied groups were compared using Pearson’s chi-squared test for categorical variables and ANOVA or Kruskal–Wallis test for continuous variables.

Multiple linear regression was used to assess associations between the categorical alcohol exposure variable and continuous BC variables with adjustments for potential confounders (age, education level, smoking status, self-reported chronic NCDs; analyses of WC and HC were additionally adjusted for height). Non-problem drinkers were the reference group in all comparisons. Results of regression analyses were presented as mean differences in BC parameters with 95% confidence intervals (95% CI). Model assumptions were assessed by visual inspection of the residual plots. STATA 17.0 (StataCorp, USA, Texas, College Station) was used for all analyses.

## 3. Results

### 3.1. Groups by Level of Alcohol Consumption

Among the participants of the main study (982 men and 1375 women), 109 (11.1%) men and 127 (9.2%) women were non-drinkers. Non-problem drinkers comprised 500 (50.9%) men and 1156 (84.1%) women, hazardous drinkers—281 (28.6%) men and 79 (5.8%) women, and harmful drinkers—92 (9.4%) men and 13 (1.0%) women. The additional sample of narcological patients consisted of 207 (76.1%) men and 65 (23.9%) women.

In drinking men, the proportions of daily drinkers among hazardous drinkers, harmful drinkers, and narcological patients were 38.4%, 39.1%, and 57.6%, respectively (Table 1). Among drinking women, daily drinking was most common in harmful drinkers (23.1%) and narcological patients (46.2%).

Among men, AUDIT scores of 8–15 were most prevalent in hazardous and harmful drinkers (74.7% and 45.7%, respectively), and scores of 16–19 among harmful drinkers and narcological patients (21.7% and 17.7%, respectively). In women, AUDIT scores of 8–15 were almost equally common in hazardous drinkers, harmful drinkers, and narcological patients (32.9%, 30.8%, and 32.3%, respectively), while scores of 16–19 were more characteristic of narcological patients (18.5%). AUDIT scores >20 were largely characteristic of men and women who were narcological patients (58.6% and 43.1%, respectively). CAGE scores of 2–3 were observed in men with hazardous drinking (57.7%), and harmful drinking (46.7%), and in narcological patients (46.9%), as well as in women with the same drinking levels (74.7%, 69.2%, and 52.3%, respectively). A CAGE score of 4 was only present in harmful drinkers (32.6% of men and 23.1% of women) and narcological patients (46.4% of men and 41.5% of women). Episodes of “zapoi” in the past 12 months were only observed in harmful drinkers (70.7% of men, 61.5% of women) and in narcological patients (82.1% of men, 81.5% of women). Other characteristics of alcohol abuse (hangover, excessive drunkenness, sleeping in clothes at night, and failing to fulfill family or other obligations because of drunkenness 2+ times per week) were only reported by harmful drinkers—4.4–5.4% of men and 7.7–15.4% of women, and by narcological patients—15.0–28.5% of men and 6.2–23.1% of women. The median annual volume of pure ethanol consumed in drinking men was highest in harmful drinkers (7.9 l/y) and narcology patients (6.2 l/y). Among women, the median annual consumption of pure ethanol was highest in harmful drinkers (5.3 l/y), twice as high as compared to narcological patients (2.4 l/y). In both men and women, there were upward trends (*p* < 0.001) in CDT (%) with increasing alcohol consumption. The highest values were in the narcological patients and the lowest were in non-drinkers.

There were differences between the studied groups in the frequencies and amounts of consumption of the three most commonly consumed types of alcohol-containing beverages (beer, wine, and spirits) (Table S1). Among men, all groups of drinkers most commonly consumed spirits: 40.4% of non-problem drinkers, 64.8% of hazardous drinkers, 54.4% of harmful drinkers, and 41.1% of narcology patients reported drinking spirits with a frequency ranging from one time per month to two times per week. Among women, non-problem drinkers were most commonly drinking wine: 23.9% reported taking wine from at least one time per month to two times per week. Hazardous, harmful drinkers, and narcological patients among women were most commonly drinking spirits in the same frequency range (38.0%, 38.5%, and 23.4%, respectively). Drinking spirits three or more times a week was most commonly reported by harmful drinkers and narcological patients among men (18.4% and 26.1%) and by narcological patients among women (23.4%). Taking five or more spirit drinks per occasion was common among harmful drinkers (63.0% of men, 32.9% of women), harmful drinkers (82.6% of men, 38.5% of women), and narcological patients (77.3% of men, 51.6% of women). At the median level, the total volume of ethanol consumed from spirits in the past 12 months was the largest compared to ethanol volumes consumed from other drinks in all groups of drinkers and both genders.

### 3.2. Socio-Demographic, Lifestyle, and Health-Related Characteristics

The mean age of the main study participants was 53.8 years. Non-drinkers and non-problem drinkers had mean ages of 55.7 and 54.7 years in men, and 56.7 and 53.8 years in women (Table 2). The mean ages of hazardous and harmful drinkers were 52.0 and 52.8 years in men, and 50.2 and 47.8 years in women. The mean age of narcological sub-study participants was 48.5 years for men and 47.9 years for women.

The largest proportions of participants who reported higher education were in non-problem drinkers in both men (43.4%) and women (41.4%). Among narcological patients, higher education was reported by 12.1% men and 15.4% women. Current smoking was most prevalent in narcological patients (84.1% in men, 75.4% in women) and least common in non-drinking men (27.5%) and in non-problem drinkers among women (12.5%). Self-reporting chronic NCDs was most common in non-drinking men (62.4%) and women (78.8%), and least common in the narcology group (44.4% in men, 56.9% in women).

Among men in the main study, healthy diet was least common in harmful drinkers (9.8%) compared to other studied subgroups (23.9–26.9%) (Table S2), while the subgroups of women from the main study did not show detectable differences on dietary quality. Among women in the main study, physical inactivity, including moderate inactivity, was most prevalent among harmful drinkers (46.2%) and least prevalent among non-drinkers (14.4%), while men from the main study with different levels of alcohol consumption did not differ in physical activity.

Among men, age-standardized means of height, weight, BMI, HC, and %FM were lower in both harmful drinkers and narcological patients compared to non-problem drinkers, while WHR and %FM in hazardous drinkers were higher compared to the same reference group (Table S3).

### 3.3. Associations of Alcohol Consumption with Body Composition

Among men, the mean BMI in harmful drinkers was lowest compared to non-problem drinkers after adjustment for age and education (−2.11), and the difference was slightly attenuated (−1.64) in the fully adjusted model (Model 4), which also included smoking and chronic diseases (Table 3). In narcological patients, compared with non-problem drinkers, the mean difference in BMI had a maximum of −3.75 with adjustments for age and education, and it was attenuated to −2.95 after all adjustments. Among women, non-drinkers had a mean BMI difference of −1.06 compared to non-problem drinkers after adjustment for age and education, while further adjustments gave the difference of −1.27. Women among narcology patients also had a lower mean BMI compared with non-problem drinkers after adjusting for age and education (−1.79) as well as after all further adjustments (−1.80).

Compared to non-problem drinkers, hazardous drinking men had on average 2.52 cm larger WC after adjustments for age, height, education, and smoking, and the difference was 2.33 after adding the adjustment for chronic diseases (Model 4). After adjustments for age, height, and education, male narcology patients had a mean WC difference of −7.88 cm compared to non-problem drinkers, and the difference reduced to −6.11 cm in the fully adjusted model. Among women, non-drinkers had a mean WC difference of −2.32 cm compared to non-problem drinkers after adjustment for age, height, and education, and the difference was −2.92 cm after further adjustments.

Among men, the mean difference in HC was −3.08 cm in harmful drinkers and −6.04 cm in narcological patients compared to non-problem drinkers after adjustment for age, height, and education, and these differences reduced to −2.26 cm and −4.68 cm, respectively, after all adjustments. In non-drinking women, the mean difference in HC was −2.07 cm in the fully adjusted model. After adjustments for age, height, and education, women who were narcological patients had a mean difference in HC of −7.05 cm compared to non-problem drinkers, and it went down to −6.36 cm with further adjustments for smoking and chronic diseases.

Mean WHR in hazardous drinking men was 0.02 higher compared to non-problem drinking men in all regression models, regardless of the adjustments. Men who were narcological patients had a mean WHR difference of −0.02 in all models with the same reference group. Among women, the mean difference in WHR was 0.04 in harmful drinkers compared to non-problem drinkers after adjustments for age and education, but it went down to being non-significant after further adjustments for smoking and chronic diseases. Women who were narcological patients had an age-adjusted mean WHR difference of 0.06 compared to non-problem drinkers, and the difference reduced to 0.03 in the fully adjusted model.

In men, the mean difference in %FM in hazardous drinkers compared to non-problem drinkers was up to 1.46% after adjustments for age, education, and smoking, and it reduced to 1.35% after the final adjustment for chronic diseases. Compared to the same reference group, harmful drinkers and narcological patients had the largest mean differences in %FM of −2.11% and −3.60%, respectively, after adjustment for age, and these differences reduced to −1.56% and −2.57% with further adjustments. Among women, the mean %FM was lower in non-drinkers compared to non-problem drinkers (−1.53%) in the fully adjusted model.

### 3.4. Supplementary Analyses

Comparisons of non-drinkers, hazardous drinkers, and harmful drinkers with non-problem drinkers in the main study (the general population sample) with additional adjustments for physical activity level and dietary quality (data were not present for narcology patients) showed minor changes in the magnitudes of the earlier presented differences in the BC parameters. Among men, the mean difference in BMI for harmful drinkers increased from −1.64 (Model 4, Table 3) to −1.73 (Table S4), the mean WC difference for hazardous drinkers reduced from 2.33 cm to 2.04 cm, the mean HC difference for hazardous drinkers increased from −2.26 cm to −2.55 cm, the mean difference in %FM for hazardous drinkers reduced from 1.35 to 1.18, and the mean %FM difference for harmful drinkers increased from −1.56 to −1.78. Among women, the mean BMI difference for non-drinkers decreased from −1.27 (Model 4, Table 3) to −1.18 (Table S4), the mean difference in WC for non-drinkers reduced from −2.92 cm to −2.69 cm, and the mean %FM difference for non-drinkers reduced from −1.53% to −1.44%.

## 4. Discussion

To the best of our knowledge, this is the first population-based study of associations between alcohol consumption and BC parameters in Russian adult men and women representing the general population and patients treated for alcohol use disorders. Hazardous alcohol consumption was found to be associated with increased adiposity-related BC parameters, but these parameters were reduced among harmful drinkers in the general population and were the lowest in patients treated for alcohol-related diagnoses.

### 4.1. Findings in the General Population Sample

Our findings of the increased adiposity-related parameters in hazardous drinkers are consistent with previous population studies, which found higher levels of BMI, WC, and WHR in heavy and binge drinkers [18,21,24,45,46,47]. They are in line with the described contributions of alcohol to normal dietary nutrients through additional energy intake from ethanol [17,43]. Alcohol consumption also causes shifts in biochemical processes with prioritized oxidation of ethanol molecules, which inhibits the oxidation of fats and carbohydrates and explains their accumulation in the body [15,48]. In addition, alcohol consumption increases appetite, and that can lead to an increase in caloric intake and weight gain [19,20]. That may also partly explain the increased BC parameters in hazardous drinkers as these findings were adjusted for dietary quality (assessed with the Dietary Quality Score [35]) but not for the quantity of the food consumed.

With respect to harmful drinkers (the group of the heaviest drinkers in the general population), our results differ from those of other studies [18,21,23,49]. Our study demonstrated that harmful drinkers had significantly lower adiposity-related BC parameters compared with non-problem and hazardous drinkers. In this sense, harmful drinkers in the general population were rather close to those treated for alcohol-related disorders, the narcology patients in our study, who had the lowest adiposity-related BC parameters among all the studied groups with varying drinking levels. The observed similarities between harmful drinkers in the general population and patients treated for alcohol-related disorders in drinking patterns, socio-demographic and behavioral characteristics, and BC parameters indicate the suitability of the categorization we used when dividing the study participants into the studied subgroups.

Our findings of the reduced BMI, WC, HC, and %FM in non-drinking women versus non-problem drinking women may be a population-specific feature. For comparison, a Scottish Health Survey and a study of adult Danes showed that frequent alcohol consumers had lower BMI and WC compared to infrequent drinkers [21,47]. Another study of French adults described an increase in WHR in both sexes and BMI in non-drinking men compared to drinkers [19]. These contradictory findings from different settings could be due to possible differences in the alcoholic beverages predominantly consumed and cultural contexts such as drinking patterns and associated eating habits.

Diet and physical activity are important contributors to shaping BC [50,51,52,53]. For this reason, we attempted to adjust the studied associations for physical activity and dietary quality, but only within the main study sample because data on physical activity and diet were unavailable for narcological patients. As the attenuations were weak, we concluded that the observed differences in BC between the compared main study groups were not explained by the varying physical activity and diet to a substantial degree. Given the described similarities between harmful drinkers and narcological patients, the unperformed corrections for diet and physical activity should not have seriously biased our findings regarding BC parameters in narcological patients.

Chronic diseases of the cardiovascular system, kidneys, liver, and cancer were also considered as potential contributors to the differences in BC between participants with different levels of drinking. The proportions of those with NCDs were highest among non-drinkers and lowest among harmful drinkers and narcological patients in both sexes. One explanation might be that heavy drinkers were healthy enough to take alcohol in larger amounts. Alternatively, they might be less aware of having NCDs because of having fewer healthcare contacts and being underdiagnosed. Moreover, their self-reports might be less reliable. Therefore, the adjustment for NCDs could have introduced some bias because of differential misreporting, and the corresponding results (Model 4, Table 3) should be interpreted with caution. This was the reason why we presented results both with and without correction for NCDs (Models 3 and 4, respectively). De facto, the adjustment for NCDs did not significantly affect our findings for drinkers of both sexes, only for non-drinking women, who were not of primary interest in this study.

Earlier studies of BC parameters measured with bioimpedance or X-ray absorptiometry showed that higher levels of drinking were positively associated with body fat percentage in both sexes [54,55]. We had similar results for hazardous drinkers but opposite results for harmful drinkers among men, and found no associations among women. The lack of significant associations in women may be explained by the small numbers of women with hazardous and harmful drinking in this study, by specific drinking patterns in women [15,45], or by the specific features of alcohol metabolism in a woman’s body [1,56].

### 4.2. Findings in the Sample of Patients Treated for Alcohol-Related Diagnoses

We found a few earlier studies of BC parameters in patients treated for alcohol-related diagnoses. Liangpunsakul et al. reported a considerable decline in BMI and %FM in chronic alcoholics, especially in men [57]. Additionally, Lenz et al. showed that standard body measurements of participants recruited from in-patient alcoholism treatment programs were significantly lower than in healthy controls for both sexes [58]. These findings correspond to our results for men, showing substantially lower BC parameters (BMI, WC, HC, WHR, %FM) in male narcological patients, compared to non-problem drinkers in the general population.

Our findings for women treated for alcohol-related disorders were more controversial and weaker due to the relatively small number of women (N = 63) among narcological patients. Nevertheless, we observed differences in selected adiposity-related BC parameters between narcological patients and non-problem drinking women from the general population, which were contrary to those in men. For instance, women among narcological patients had a smaller and insignificant difference in WC compared to non-problem drinkers, while there was a substantial difference in men. Conversely, women treated for alcohol problems had a more substantially reduced mean HC, compared to the HC reduction observed in men in the same treatment. That resulted in a higher WHR among the narcological patients vs non-problem drinkers in women, which was the opposite of what we saw in men. This could mean that severely drinking men were becoming thinner in waist to a greater degree than in hips, while the severely drinking women did not get much thinner in waist but were to a larger degree thinner in hips. These differences could be explained by women being generally thinner in the waist compared with men, so heavy drinking could lead to more fat losses on women’s hips, where it is more commonly deposited. However, we cannot go beyond hypothesizing on this matter based on what we have observed in a cross-sectional study. The finding requires further investigation with a longitudinal approach and an emphasis on physiological differences in alcohol metabolism in men and women.

Despite our efforts to control for dietary quality, the observed lower BC parameters in narcological patients and harmful drinkers may be partially attributed to reduced food consumption associated with binge drinking. This phenomenon may be explained by changes in the brain reward system and the associated neurohumoral imbalances [49], resulting in an increased threshold for satisfying needs and addiction [59]. This can manifest itself as changes in eating and social behavior and in long periods of binge drinking with inadequate nutrition [60] when food and non-alcoholic beverage intake decreases [22,61]. The decreased BC parameters may also be explained by other effects of alcohol, such as malabsorption [62], hepatotoxicity [63], hormonal imbalances [64], oxidative stress [65], alcohol interactions with medication [66], increased urine output [67,68,69], and alcohol-induced diarrhea [70,71]. Lastly, although our findings were adjusted for education, other social and economic factors such as access to healthy food and knowledge about healthy nutrition could also play a role [72].

The described BC distortions in narcological patients require clinical attention as they may exacerbate the negative health effects of alcohol. Poor nutritional status can lead to a weakened immune system, increased susceptibility to infections [73], and increased risks of cardiovascular disease, type 2 diabetes, cancer [74], cognitive impairment, depression, and other mental health disorders [75,76]. Additionally, muscle wasting and weakness can impede physical performance, increase risks of injuries, and negatively affect the quality of life [77]. Therefore, BC control and nutritional management should be a component of treatment and rehabilitation for patients with alcohol-related disorders [78].

An unexpected observation regarding narcological patients was the lower median volume of annual ethanol consumed compared to harmful drinking men and women from the general population. This could be due to the preceding treatment periods when the patients were abstaining from alcohol. That was unlikely to be a problem as one of the criteria used for patients’ selection was a documented history of alcohol-related problems.

Other social and lifestyle characteristics of patients with alcohol use disorders in our study are consistent with prior research, which have shown that people with chronic alcohol abuse have lower education levels and a large proportion are smokers [79,80].

### 4.3. Limitations

In our study, we compared BC parameters in subgroups of participants from the general population with different levels of alcohol consumption. Group allocation was based on the self-reported alcohol consumption characteristics in the past 12 months and the results of two tests for the identification of alcohol abuse: AUDIT and CAGE. Self-reports and test results are subjective and could be prone to recall bias, which is typical of alcohol studies [81]. Participants could also provide socially desirable answers, thus introducing a response bias. Therefore, the scores of the AUDIT and CAGE tests, as well as self-reported harmful drinking indicators, could be biased due to underreporting and recall problems. These deficiencies could lead to misclassifications in the allocation of participants into the studied subgroups by alcohol consumption levels. To substantiate the group division, we compared them on several alcohol-related parameters, including the total annual ethanol consumed over the preceding 12 months, estimated using the quantity–frequency method [29] and the level of %CDT as a marker of drinking in the past 14 days. The observed consistency in the growth of these parameters along with the increase in the group order provided grounds to conclude that the division of participants into groups was reasonably valid. These findings also supported the validity of the used categorization in a broader perspective.

A deficiency in the criteria used for group allocation resulted in a considerable proportion of the study population being categorized as non-problem drinkers. Other groups were relatively small, thus limiting the statistical power of the comparisons. Moreover, the classification criteria were uniform for men and women, and therefore, not many women were categorized as hazardous (N = 79) or harmful drinkers (N = 13), and there was a small number of women among the narcological patients (N = 63). This further limited the power to detect differences between these groups and non-problem drinkers among women.

Another limitation is that we lacked data to divide the group of non-drinkers into lifelong never-drinkers and those who drank in the past. As presented in the results, non-drinkers in both men and women more commonly reported having chronic diseases compared to other studied groups, possibly indicating that non-drinkers included quitters because of health problems. This could have biased our findings regarding non-drinkers, which means the results require cautious interpretation. This deficiency can be treated as insubstantial because non-drinkers were not the focus of this study.

The percentage of body fat mass in our study was measured using the TANITA BC-418 device, which functions based on a modified 8-electrode bioimpedance method. This method provided a distorted view of BC, as, for example, described in the study of Veronica Luque et al. [82]. It is also worth mentioning that some of the recommendations of the manufacturer for accurate measurement were not strictly implemented, and that could have affected the accuracy and reliability of the results obtained. These recommendations included abstaining from alcohol and vigorous exercise for at least 12 h prior to measurement, refraining from urinating immediately before measurement, and not taking measurements in women during their menstrual period [41]. However, our findings regarding BC parameters measured with the bioimpedance method were in line with findings from conventional anthropometric measurements. This consistency between the two types of measurements suggests the validity of the findings was not substantially impaired.

A deeper understanding of the effects of alcohol on BC parameters could have been achieved with this study if the available data included laboratory measurements of serum biomarkers and metabolic active products of adipose tissue lipocytes: growth factors (FGF-21 and FGF-23 [83], VEGF [84], TGF-β [85]), lipid acids [84], adipokines (adiponectin, leptin), cytokines (IL-1β, IL-8, IL-10, IL-18, IL-17D, MCP-1), prostaglandins, extracellular matrix (collagen II-VI, thrombospondin-1) [85], and inhibitors of proteases (cystatin C [86], TIMP-1 [87]). Based on this limitation, investigation of the metabolic mechanisms underlying the described associations is a prospect for future studies on the topic.

The response rate of 68% in the main part of the KYH study in Arkhangelsk could be a source of a sampling bias if the attendance was dependent on the strength of the association between BC parameters and alcohol use. That would be, for example, if obese hazardous drinkers were more willing to participate in the study compared to individuals with other combinations of alcohol and BC characteristics.

Finally, the cross-sectional design of the study limited our conclusions to the statements of the associations, without causality proofs.

## 5. Conclusions

Alcohol consumption was found to have an inverted J-shaped relationship with adiposity-related body composition parameters among men in the general population. These parameters were elevated in hazardous drinkers but were reduced in harmful drinkers and were even lower in patients with alcohol-related diagnoses, compared to non-problem drinking men in the general population. Heavy drinking women who were in-patients treated for alcohol problems had a reduced hips circumference but not waist circumference, which resulted in a higher waist-to-hip ratio compared to non-problem drinking women in the general population. Prospective studies and in-depth investigations of underlying metabolic mechanisms may shed more light on the effects of alcohol exposure on the human body.

## Figures and Tables

**Table 1:** Alcohol consumption characteristics in the last 12 months in the general population sample divided by the level of alcohol consumption and in the additional sample of patients treated for alcohol use disorders, by sex.

Characteristics	Men (N = 1189)					Women (N = 1440)				
	Non-Drinkers	Non-Problem Drinkers	Hazardous Drinkers	Harmful Drinkers	Narcology	Non-Drinkers	Non-Problem Drinkers	Hazardous Drinkers	Harmful Drinkers	Narcology
N	109	500	281	92	207	127	1156	79	13	65
Frequency of alcohol use, N (%)										
Never or rarely	109 (100)	187 (37.4)	35 (12.5)	18 (19.6)	34 (16.6)	127 (100)	751 (65.0)	21 (26.6)	5 (38.5)	20 (30.8)
1–5 times per month	—	234 (46.8)	138 (49.1)	38 (41.30)	53 (25.9)	—	373 (32.3)	49 (62.0)	5 (38.5)	15 (23.1)
Nearly every day	—	79 (15.8)	108 (38.4)	36 (39.1)	118 (57.6)	—	31 (2.3)	9 (11.4)	3 (23.1)	30 (46.2)
AUDIT score categories, N (%)										
<7	109 (100)	497 (100)	71 (25.3)	17 (18.5)	3 (1.5)	127 (100)	1154 (100)	53 (67.1)	7 (53.9)	4 (6.2)
8–15	—	—	210 (74.7)	42 (45.7)	45 (22.2)	—	—	26 (32.9)	4 (30.8)	21 (32.3)
16–19	—	—	—	20 (21.7)	36 (17.7)	—	—	—	1 (7.7)	12 (18.5)
>20	—	—	—	13 (14.1)	119 (58.6)	—	—	—	1 (7.7)	28 (43.1)
CAGE score categories, N (%)										
<2	500 (100)	109 (100)	119 (42.4)	19 (20.7)	14 (6.8)	127 (100)	1156 (100)	20 (25.3)	1 (7.7)	4 (6.2)
2–3	—	—	162 (57.7)	43 (46.7)	97 (46.9)	—	—	59 (74.7)	9 (69.2)	34 (52.3)
4	—	—	—	30 (32.6)	96 (46.4)	—	—	—	3 (23.1)	27 (41.5)
“Zapoi”, ≥1 episode	—	—	—	65 (70.7)	170 (82.1)	—	—	—	8 (61.5)	53 (81.5)
Hangover 2+ times/week	—	—	—	4 (4.4)	59 (28.5)	—	—	—	2 (15.4)	15 (23.1)
Excessive drunkenness 2+ times/week	—	—	—	4 (4.4)	38 (18.4)	—	—	—	1 (7.7)	11 (16.9)
Sleeping in clothes because of drunkenness 2+ times/week	—	—	—	4 (4.4)	31 (15.0)	—	—	—	—	4 (6.2)
Failing to fulfil family or other obligations 2+ times/week	—	—	—	5 (5.4)	36 (17.4)	—	—	—	2 (15.4)	12 (18.5)
Pure ethanol consumption per year, l/year, Me (P25–P75)	—	1.9 (0.7; 4.8)	7.0 (2.8; 14.1)	7.9 (3.3; 20.8)	6.2 (2.3; 19.7)	—	0.5 (0.2; 1.2)	2.8 (1.3; 6.0)	5.3 (2.8; 21.5)	2.4 (1.7; 12.9)
CDT (%), Me (P25; P75) *	0.6 (0.5; 0.7)	0.8 (0.6; 1.0)	0.9 (0.7; 1.4)	1.1 (0.7; 2.8)	1.8 (1.0; 2.9)	0.4 (0.4; 0.6)	0.7 (0.5; 0.8)	0.7 (0.6; 0.9)	0.7 (0.5; 0.9)	1.2 (0.8; 1.9)

* Carbohydrate-deficient transferrin (CDT) data were available for a subsample of 1242 KYH participants (74 non-drinkers, 446 non-problem drinkers, 359 hazardous drinkers, 97 harmful drinkers, 266 narcology patients); *p*-value for trend < 0.001 for both men and women.

**Table 2:** Socio-demographic, lifestyle, and health-related characteristics of the studied groups divided by annual alcohol intake and the group of narcological patients, by sex.

Characteristics	Men (N = 1189)						Women (N = 1440)					
	Non-Drinkers	Non-Problem Drinkers	Hazardous Drinkers	Harmful Drinkers	Narcology	*p*-Value *	Non-Drinkers	Non-Problem Drinkers	Hazardous Drinkers	Harmful Drinkers	Narcology	*p*-Value *
N	109	500	281	92	207		127	1156	79	13	65	
Age at health check (years), Mean ± SD	55.7 ± 9.4	54.7 ± 9.7	52.0 ± 9.3	52.8 ± 9.3	48.5 ± 8.5	< 0.001	56.7 ± 10.0	53.8 ± 9.8	50.2 ± 8.7	47.8 ± 6.9	47.9 ± 9.0	<0.001
Education, N (%)						< 0.001						<0.001
Primary	18 (16.5)	35 (7.00)	28 (10.0)	8 (8.7)	53 (25.6)		15 (11.8)	60 (5.2)	8 (10.1)	1 (7.7)	14 (21.5)	
Secondary	62 (56.9)	248 (49.6)	149 (53.0)	64 (69.6)	129 (62.3)		72 (56.7)	618 (53.5)	46 (58.2)	10 (76.9)	41 (63.1)	
Higher	29 (26.6)	217 (43.4)	104 (37.0)	20 (21.7)	25 (12.1)		40 (31.5)	478 (41.4)	25 (31.7)	2 (15.4)	10 (15.4)	
Smoking status, N (%)						< 0.001						<0.001
Never a smoker	32 (29.4)	181 (36.2)	70 (24.9)	5 (5.4)	16 (7.7)		86 (67.7)	848 (73.4)	32 (40.5)	1 (7.7)	12 (18.5)	
Ex-smoker	47 (43.1)	174 (34.8)	105 (37.4)	29 (31.5)	17 (8.2)		19 (15.0)	163 (14.1)	15 (19.0)	2 (15.4)	4 (6.2)	
Current smoker	30 (27.5)	145 (29.0)	106 (37.7)	58 (63.0)	174 (84.1)		22 (17.3)	145 (12.5)	32 (40.5)	10 (76.9)	49 (75.4)	
Presence of chronic diseases **, N (%)	68 (62.4)	288 (57.6)	162 (57.7)	48 (52.2)	92 (44.4)	0.007	99 (78.0)	743 (64.3)	51 (64.6)	9 (69.2)	37 (56.9)	0.020

* *p*-value from ANOVA for numerical variables, and Pearson’s chi-squared test for categorical variables. ** Any of the following: cardiovascular disease (hypertension, angina, myocardial infarction, stroke, heart failure, or atrial fibrillation), cancer, chronic liver disease, chronic kidney disease.

**Table 3:** Associations of alcohol consumption levels with body composition parameters in men and women.

Outcome	Men (N = 1189)				Women (N = 1440)			
	Model 1 ^a^	Model 2 ^b^	Model 3 ^c^	Model 4 ^d^	Model 1 ^a^	Model 2 ^b^	Model 3 ^c^	Model 4 ^d^
BMI, B (95% CI)								
Non-problem drinkers	Ref.	Ref.	Ref.	Ref.	Ref.	Ref.	Ref.	Ref.
Non-drinkers	−0.17 (−1.09; 0.74)	−0.18 (−1.10; 0.74)	−0.28 (−1.19; 0.63)	−0.32 (−1.21; 0.58)	−0.94 (−1.98; 0.10)	−1.06 (−2.09; −0.02)	−1.06 (-2.10; −0.02)	−1.27 (−2.29; −0.26)
Hazardous drinkers	0.43 (−0.21; 1.08)	0.43 (−0.22; 1.08)	0.50 (−0.14; 1.15)	0.43 (−0.21; 1.07)	0.06 (−1.23; 1.35)	−0.13 (−1.42; 1.15)	−0.13 (−1.44; 1.17)	−0.30 (−1.58; 0.99)
Harmful drinkers	−2.10 (−3.08; −1.12)	−2.11 (−3.10; −1.12)	−1.67 (−2.66; −0.67)	−1.64 (−2.63; −0.66)	1.74 (−1.35; 4.83)	1.34 (−1.74; 4.43)	1.37 (-1.76; 4.49)	1.00 (−2.06; 4.06)
Narcology patients	−3.74 (−4.48; −3.00)	−3.75 (−4.52; −2.98)	−2.94 (−3.74; −2.13)	−2.95 (−3.74; −2.16)	−1.30 (−2.72; 0.12)	−1.79 (−3.23; −0.36)	−1.75 (−3.27; −0.24)	−1.80 (−3.28; −0.31)
WC (cm), B (95% CI) ^e^								
Non-problem drinkers	Ref.	Ref.	Ref.	Ref.	Ref.	Ref.	Ref.	Ref.
Non-drinkers	0.44 (−1.93; 2.81)	0.31 (−2.07; 2.70)	0.01 (−2.34; 2.36)	−0.07 (−2.39; 2.24)	−1.98 (−4.42; 0.45)	−2.32 (−4.75; −0.10)	−2.41 (−4.84; 0.02)	−2.92 (−5.30; −0.54)
Hazardous drinkers	2.49 (0.81; 4.16)	2.43 (0.75; 4.11)	2.52 (0.86; 4.19)	2.33 (0.69; 3.96)	1.08 (−1.95; 4.11)	0.51 (−2.50; 3.53)	0.19 (−2.87; 3.25)	−0.20 (−3.20; 2.79)
Harmful drinkers	−2.41 (−4.96; 0.13)	−2.54 (−5.10; 0.02)	−1.68 (−4.26; 0.90)	−1.59 (−4.13; 0.95)	7.13 (−0.13; 14.38)	6.02 (−1.19; 13.24)	5.33 (−1.98; 12.64)	4.44 (−2.72; 11.60)
Narcology patients	−7.62 (−9.55; −5.68)	−7.88 (−9.89; −5.87)	−6.12 (−8.22; −4.03)	−6.11 (−8.18; −4.05)	−0.05 (−3.39; 3.30)	−1.45 (−4.82; 1.92)	−2.05 (−5.60; 1.50)	−2.18 (−5.65; 1.30)
HC (cm), B (95% CI) ^e^								
Non-problem drinkers	Ref.	Ref.	Ref.	Ref.	Ref.	Ref.	Ref.	Ref.
Non-drinkers	0.53 (−1.07; 2.13)	0.52 (−1.10; 2.13)	0.35 (−1.24; 1.94)	0.31 (−1.27; 1.89)	−1.64 (−3.64; 0.35)	−1.83 (−3.82; 0.17)	−1.75 (−3.75; 0.24)	−2.07 (−4.05; −0.09)
Hazardous drinkers	0.54 (−0.60; 1.67)	0.53 (−0.61; 1.66)	0.67 (−0.45; 1.80)	0.58 (−0.54; 1.70)	−0.47 (−2.96; 2.01)	−0.77 (−3.25; 1.71)	−0.43 (−2.95; 2.08)	−0.67 (−3.16; 1.82)
Harmful drinkers	−3.07 (−4.79; −1.35)	−3.08 (−4.82; −1.35)	−2.30 (−4.04; −0.56)	−2.26 (−3.99; −0.53)	2.18 (−3.76; 8.12)	1.63 (−4.31; 7.56)	2.42 (−3.59; 8.44)	1.88 (−4.07; 7.83)
Narcology patients	−6.00 (−7.32; −4.69)	−6.04 (−7.40; −4.68)	−4.68 (−6.10; −3.26)	−4.68 (−6.08; −3.27)	−6.31 (−9.05; −3.57)	−7.05 (−9.83; −4.28)	−6.29 (−9.21; −3.37)	-6.36 (−9.25; −3.47)
WHR, B (95% CI)								
Non-problem drinkers	Ref.	Ref.	Ref.	Ref.	Ref.	Ref.	Ref.	Ref.
Non-drinkers	−0.00 (−0.01; 0.01)	−0.00 (−0.02; 0.01)	−0.00 (−0.02; 0.01)	−0.00 (−0.02; 0.01)	−0.01 (−0.02; 0.01)	−0.01 (−0.02; 0.00)	−0.01 (−0.02; 0.00)	−0.01 (−0.02; 0.00)
Hazardous drinkers	0.02 (0.01; 0.03)	0.02 (0.01; 0.03)	0.02 (0.01; 0.03)	0.02 (0.01; 0.03)	0.01 (−0.00; 0.03)	0.01 (−0.00; 0.03)	0.01 (−0.01; 0.02)	0.00 (−0.01; 0.02)
Harmful drinkers	0.00 (−0.01; 0.02)	0.00 (−0.01; 0.02)	0.00 (−0.01; 0.02)	0.00 (−0.01; 0.02)	0.05 (0.01; 0.09)	0.04 (0.01; 0.08)	0.03 (−0.01; 0.07)	0.03 (−0.01; 0.06)
Narcology patients	−0.02 (−0.03; −0.01)	−0.02 (−0.03; −0.01)	−0.02 (−0.03; −0.01)	−0.02 (−0.03; −0.01)	0.06 (0.04; 0.07)	0.05 (0.03; 0.07)	0.04 (0.02; 0.05)	0.03 (0.02; 0.05)
%FM (%), B (95% CI)								
Non-problem drinkers	Ref.	Ref.	Ref.	Ref.	Ref.	Ref.	Ref.	Ref.
Non-drinkers	−0.49 (−1.87; 0.90)	−0.46 (−1.86; 0.93)	−0.61 (−1.99; 0.77)	−0.67 (−2.03; 0.70)	−1.26 (−2.61; 0.09)	−1.32 (−2.68; 0.03)	−1.29 (−2.65; 0.06)	−1.53 (−2.86; −0.19)
Hazardous drinkers	1.41 (0.43; 2.39)	1.42 (0.44; 2.41)	1.46 (0.49; 2.44)	1.35 (0.39; 2.32)	0.21 (−1.46; 1.89)	0.09 (−1.58; 1.77)	0.25 (−1.45; 1.95)	0.07 (−1.60; 1.75)
Harmful drinkers	−2.11 (−3.59; −0.62)	−2.06 (−3.56; −0.56)	−1.59 (−3.10; −0.08)	−1.56 (−3.04; −0.07)	3.03 (−0.97; 7.04)	2.75 (−1.26; 6.77)	3.17 (−0.89; 7.24)	2.76 (−1.24; 6.77)
Narcology patients	−3.60 (−4.71; −2.49)	−3.56 (−4.72; −2.39)	−2.56 (−3.78; −1.34)	−2.57 (−3.77; −1.37)	−1.57 (−3.43; 0.28)	−1.86 (−3.75; 0.02)	−1.42 (−3.40; 0.56)	−1.49 (−3.44; 0.46)

Abbreviation: body mass index (BMI), hip circumference (HC), waist circumference (WC), waist-to-hip ratio (WHR). ^a^ Model 1 adjusted for age; ^b^ Model 2 adjusted as per Model 1 plus education; ^c^ Model 3 adjusted as per Model 2 plus smoking status; ^d^ Model 4 adjusted as per Model 3 plus a self-reported chronic disease (cardiovascular diseases, cancer, liver diseases, kidney diseases). ^e^ Models 1–4 for HC and WC are additionally adjusted for height (cm).

## Data Availability

Data from the Know Your Heart study are available upon reasonable request with permission of the Know Your Heart Steering Group. See data access regulations and instructions at https://metadata.knowyourheart.science (accessed on 30 December 2022). All data requests will be guided by the protection of personal information, the confidentiality agreement with the participants, and participants’ informed consent.

## Supplementary Materials

The following supporting information can be downloaded at: https://www.mdpi.com/article/10.3390/ijerph20042905/s1, Table S1. Types of alcoholic beverages consumed in the last 12 months in the sample of drinkers from the general population divided by total annual alcohol intake and in patients treated for alcohol-related disorders, by sex; Table S2. Dietary quality and physical activity categories in the general population groups* defined by annual alcohol intake, by sex; Table S3. Age-standardized means with 95% Confidence Intervals* of body composition parameters in the general population groups defined by annual alcohol intake and in the group of narcological patients, by sex; Table S4. Associations of the alcohol consumption levels with body composition parameters in men and women from the general population sample with additional adjustments for dietary quality and physical activity^*^, B-coefficients with 95% Confidence Intervals.
