# Supplementary material for: Association between Alcohol Consumption and Body Composition in Russian Adults and Patients Treated for Alcohol-Related Disorders: The Know Your Heart Cross-Sectional Study
Source: Int J Environ Res Public Health. 2023 Feb 7;20(4):2905. doi: 10.3390/ijerph20042905 (PMC9957482; doi:10.3390/ijerph20042905)
Supplement: Supplementary file 1 [file ijerph-20-02905-s001.zip › ijerph-2163826-supplementary.pdf]

Supplementary Materials

Table S1. Types of alcoholic beverages consumed in the last 12 months in the sample of drinkers from the general population divided by total annual alcohol intake and in patients treated for alcohol-related disorders, by sex.

Study variables	Drinking men				Drinking women			
	Non-problem drinkers	Hazardous drinkers	Harmful drinkers	Narcology	Non-problem drinkers	Hazardous drinkers	Harmful drinkers	Narcology
Frequency of beer drinking, N (%)								
A few times per year or never	356 (71.2)	164 (58.4)	55 (59.8)	142 (68.6)	1058 (91.5)	66 (83.5)	8 (61.5)	49 (76.6)
1 time per month – 2 times per week	133 (26.6)	93 (33.1)	29 (31.5)	39 (18.8)	97 (8.4)	13 (16.5)	3 (23.1)	9 (14.1)
3 or more times per week	11 (2.2)	24 (8.5)	8 (8.7)	26 (12.6)	1 (0.1)	—	2 (15.4)	6 (9.3)
Frequency of wine drinking, N (%)								
A few times per year or never	394 (78.8)	211 (75.1)	74 (80.4)	165 (80.5)	875 (75.7)	47 (59.5)	10 (76.9)	49 (75.4)
1 time per month – 2 times per week	92 (18.4)	58 (20.6)	12 (13.0)	28 (13.7)	276 (23.9)	28 (35.4)	2 (15.4)	14 (21.5)
3 or more times per week	14 (2.8)	12 (4.3)	6 (6.6)	12 (5.9)	5 (0.4)	4 (5.1)	1 (7.7)	2 (3.1)
Frequency of spirits drinking, N (%)								
A few times per year or never	271 (54.2)	65 (23.1)	25 (27.2)	68 (32.8)	998 (86.3)	49 (62.0)	7 (53.9)	34 (53.1)
1 time per month – 2 times per week	202 (40.4)	182 (64.8)	50 (54.4)	85 (41.1)	153 (13.2)	30 (38.0)	5 (38.5)	15 (23.4)
3 or more times per week	27 (5.4)	34 (12.1)	17 (18.4)	54 (26.1)	5 (0.4)	—	1 (7.7)	15 (23.4)
Amount of beer consumption per occasion, N (%)								
Never drinks beer	277 (55.4)	135 (48.0)	41 (44.6)	126 (61.2)	904 (78.2)	52 (65.8)	8 (61.5)	44 (67.7)
1 bottle (0.5l) or less	121 (24.2)	34 (12.1)	21 (22.8)	58 (28.2)	202 (17.5)	8 (10.1)	—	15 (23.1)
2-4 bottles	97 (19.4)	99 (35.2)	21 (22.8)	16 (7.8)	50 (4.3)	18 (22.8)	4 (30.8)	3 (4.6)
More than 5 bottles	5 (1.0)	13 (4.6)	9 (9.8)	6 (2.9)	—	1 (1.3)	1 (7.7)	3 (4.6)
Amount of wine consumption per occasion, N (%)								
Never drinks wine	281 (56.2)	171 (60.9)	59 (64.1)	146 (71.9)	388 (33.6)	29 (36.7)	8 (61.5)	37 (57.8)
1 glass (0.2l) or less	87 (17.4)	24 (8.5)	8 (8.7)	8 (3.9)	416 (36.0)	9 (11.4)	—	8 (12.5)
2-4 glasses	125 (25.0)	70 (24.9)	15 (16.3)	34 (16.8)	346 (29.9)	35 (43.0)	4 (30.8)	17 (26.6)
More than 5 glasses	7 (1.4)	16 (5.7)	10 (10.9)	15 (7.4)	6 (0.5)	7 (8.9)	1 (7.7)	2 (3.1)
Amount of spirits consumption per occasion, N (%)								
Never drinks spirits	83 (16.6)	17 (6.1)	2 (2.2)	4 (1.9)	626 (54.2)	24 (30.4)	5 (38.5)	12 (18.8)
1 shot (0.05l) or less	34 (6.8)	4 (1.4)	—	4 (1.9)	143 (12.4)	—	1 (7.7)	1 (1.6)
2-4 shots	241 (48.7)	83 (29.5)	14 (15.2)	39 (18.9)	345 (29.8)	29 (36.7)	2 (15.4)	18 (28.1)
More than 5 shots	139 (27.9)	177 (63.0)	76 (82.6)	159 (77.3)	42 (3.6)	26 (32.9)	5 (38.5)	33 (51.6)
Annual volume of ethanol from beer, l/year, Me (P25; P75)	0.0 (0.0; 0.7)	0.1 (0; 2.0)	0.1 (0; 1.6)	0.0 (0; 0.68)	0.0 (0.0; 0.0)	0.0 (0.0; 0.3)	0.0 (0.0; 5.3)	0.0 (0.0; 0.1)
Annual volume of ethanol from wine, l/year, Me (P25; P75)	0.0 (0.0; 0.5)	0.0 (0.0; 0.7)	0.0 (0.0; 0.7)	0.0 (0.0; 0.2)	0.2 (0.0; 0.5)	0.5 (0.0; 1.2)	0.0 (0.0; 0.9)	0.0 (0.0; 0.7)
Annual volume of ethanol from spirits, l/year, Me (P25; P75)	1.2 (0.4; 2.8)	4.0 (1.7; 9.2)	5.1 (2.8; 12.9)	5.1 (1.7; 16.6)	0.0 (0.0; 0.4)	1.2 (0.0; 2.8)	1.1 (0.0; 2.8)	1.7 (0.7; 12.9)

Table S2. Dietary quality and physical activity categories in the general population groups * defined by annual alcohol intake, by sex.

	Men (N = 1189)					Women (N = 1440)				
	Non-drinkers	Non-problem drinkers	Hazardous drinkers	Harmful drinkers	p-value**	Non-drinkers	Non-problem drinkers	Hazardous drinkers	Harmful drinkers	p-value**
N	109	500	281	92		127	1156	79	13	
Dietary quality, N (%)					0.048					0.432
Unhealthy diet	6 (5.6)	25 (5.0)	20 (7.1)	7 (7.6)		9 (7.1)	47 (4.1)	6 (7.6)	1 (7.6)	
Average diet	73 (67.6)	345 (69.4)	194 (69.0)	76 (82.6)		74 (58.7)	689 (59.8)	49 (62.0)	6 (46.2)	
Healthy diet	29 (26.9)	127 (25.6)	67 (23.9)	9 (9.8)		43 (34.1)	416 (36.1)	24 (30.4)	6 (46.2)	
Physical activity, N (%)					0.429					0.027
Inactive	4 (3.7)	25 (5.0)	16 (5.7)	6 (6.5)		5 (4.0)	87 (7.6)	3 (3.8)	2 (15.4)	
Moderately inactive	8 (7.3)	54 (10.9)	27 (9.7)	12 (13.0)		13 (10.4)	110 (9.6)	8 (10.1)	4 (30.8)	
Moderately active	56 (51.4)	279 (56.1)	138 (49.5)	47 (51.1)		79 (63.2)	673 (58.4)	42 (53.2)	2 (15.4)	
Active	41 (37.6)	139 (28.0)	98 (35.1)	27 (29.4)		28 (22.4)	282 (24.5)	26 (32.9)	5 (38.4)	

* Data on physical activity and dietary quality was not available for the group of patients treated for alcohol-related diagnoses (narcological patients). ** p-value from Pearson chi-squared test.

Table S3. Age-standardized means with 95% Confidence Intervals * of body composition parameters in the general population groups defined by annual alcohol intake and in the group of narcological patients, by sex.

Characteristics	Men (N = 1189)					Women (N = 1440)				
	Non-drinkers	Non-problem drinkers	Hazardous drinkers	Harmful drinkers	Narcology group	Non-drinkers	Non-problem drinkers	Hazardous drinkers	Harmful drinkers	Narcology group
N	109	500	281	92	207	127	1156	79	13	65
Height (cm)	173.9 (172.8; 175.1)	176.2 (175.6; 176;8)	175.7 (175.0; 176.4)	**173.9 (172.6; 175.1)**	**172.7 (171.7; 173.6)**	161.0 (159.7; 162.3)	162.1 (161.8; 162.4)	162.0 (160.8; 163.3)	163.9 (162.2; 165.6)	160.9 (159.8; 161.9)
Weight (kg)	82.8 (79.5; 86.1)	85.4 (84.0; 86.9)	86.2 (84.4; 87.9)	**76.8 (74.1; 79.6)**	**71.7 (69.6; 73.9)**	71.1 (67.6; 74.6)	72.8 (71.9; 73.7)	72.9 (69.8; 76.0)	81.2 (75.5; 86.9)	68.9 (65.5; 72.3)
BMI (kg/m^2)	27.3 (26.4; 28.2)	27.5 (27.1; 27.9)	27.9 (27.4; 28.4)	**25.4 (24.5; 26.2)**	**24.0 (23.4; 24.6)**	27.4 (26.2; 28.6)	27.7 (27.4; 28.1)	27.7 (26.6; 28.8)	30.6 (28.3; 33.0)	26.6 (25.4; 27.8)
WC (cm)	94.3 (91.7; 97.0)	94.8 (93.8; 95.9)	97.1 (95.7; 98.4)	91.6 (89.2; 93.9)	**86.3 (84.3; 88.4)**	86.1 (83.0; 89.2)	87.1 (86.4; 87.9)	88.3 (85.6; 91.0)	95.5 (89.5; 101.5)	87.3 (84.7; 90.0)
HC (cm)	101.0 (99.2; 102.7)	101.3 (100.6; 102.1)	101.6 (100.7; 102.5)	**97.1 (95.7; 98.6)**	**94.2 (92.9; 95.4)**	103.7 (101.3; 106.2)	104.6 (104.0; 105.3)	103.6 (101.3; 106.0)	109.7 (106.4; 113.1)	**98.7 (96.2; 101.3)**
WHR	0.93 (0.92; 0.95)	0.93 (0.93; 0.94)	**0.95 (0.95; 0.96)**	0.94 (0.93; 0.95)	0.91 (0.90; 0.93)	0.83 (0.81; 0.84)	0.83 (0.83; 0.83)	0.85 (0.83; 0.87)	0.86 (0.83; 0.90)	0.88 (0.87; 0.89)
%Fat mass	21.8 (20.3; 23.3)	22.2 (21.6; 22.8)	**23.6 (22.9; 24.3)**	**20.0 (18.6; 21.4)**	**19.0 (17.9; 20.2)**	33.7 (32.1; 35.3)	34.6 (34.2; 35.0)	34.7 (33.2; 36.2)	37.9 (33.8; 42.1)	33.2 (31.1; 35.3)

Abbreviation: Body Mass Index (BMI), Body Weight (BW), Hip Circumference (HC), Waist Circumference (WC), Waist-to-hip ratio (WHR). * Data are standardized to the Standard European Population 2013.

Table S4. Associations of the alcohol consumption levels with body composition parameters in men and women from the general population sample with additional adjustments for dietary quality and physical activity *, B-coefficients with 95% Confidence Intervals.

Drinker group	BMI	WC	HC	WHR	%FM
Men					
Non-problem drinkers	Ref.	Ref.	Ref.	Ref.	Ref.
Non-drinkers	-0.30 (-1.23; 0.63)	-0.06 (-2.46; 2.34)	-0.37 (-1.23; 1.98)	-0.01 (-0.02; 0.01)	-0.63 (-1.99; 0.73)
Hazardous drinkers	0.31 (-0.35; 0.97)	**2.04 (0.34; 3.73)**	0.45 (-0.69; 1.59)	**0.02 (0.01; 0.02)**	**1.18 (0.21; 2.14)**
Harmful drinkers	**-1.73 (-2.75; -0.70)**	-1.99 (-4.62; 0.64)	**-2.55 (-4.32; -0.79)**	0.00 (-0.01; 0.02)	**-1.78 (-3.27; -0.29)**
Women					
Non-problem drinkers	Ref.	Ref.	Ref.	Ref.	Ref.
Non-drinkers	**-1.18 (-2.20; -0.15)**	**-2.69 (-5.09; -0.28)**	-1.91 (-3.91; -0.08)	-0.01 (-0.02; 0.00)	**-1.44 (-2.76; -0.11)**
Hazardous drinkers	-0.26 (-1.54; 1.01)	-0.16 (-3.16; 2.84)	-0.61 (-3.09; 1.87)	0.00 (-0.01; 0.02)	0.13 (-1.52; 1.77)
Harmful drinkers	0.40 (-2.65; 3.46)	3.23 (-3.94; 10.41)	0.88 (-5.05; 6.82)	0.02 (-0.02; 0.06)	2.04 (-1.90; 5.98)

Abbreviation: Body Mass Index (BMI), Body Weight (BW), Hip Circumference (HC), Waist Circumference (WC), Waist-to-hip ratio (WHR), fat-mass percentage (%FM)/. *Adjusted as per Model 4 in Table 3 plus dietary quality (unhealthy, average, healthy) and physical activity level (inactive, moderately inactive, moderately active, active).
